# Supplementary material for: Discovery of first-in-class inhibitors of the TRF1:TIN2 protein:protein interaction by fragment screening
Source: Sci Rep. 2025 Nov 20;15:40922. doi: 10.1038/s41598-025-23858-3 (PMC12635197; doi:10.1038/s41598-025-23858-3)
Supplement: Supplementary file 2 — Supplementary Material 2 [file 41598_2025_23858_MOESM2_ESM.pdf]

Supplementary Information for

Discovery of first-in-class inhibitors of the TRF1:TIN2 protein:protein interaction by fragment screening

Giacomo Casale, Manjuan Liu, Yann-Vaï Le Bihan, Oviya Inian, Ellie Stammers, John Caldwell, Rob L. M. van Montfort, Ian Collins, Sebastian Guettler

Supplementary Table 1: Analysis of commercially available analogues of XChem screen hit 1.

Compound no.	Structure	Aq. Sol. (μM)	CPMG reduction (%)[a]	CPMG recovery (%)[b]	Crystal structure
**20**	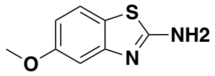	580	25	0	Yes
**21**	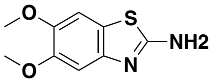	190	14	12	No
**22**	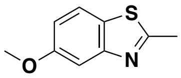	790	18	9	Yes
**23**	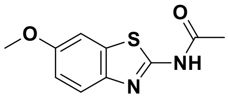	150	32	10	No
**24**	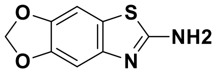	400	28	11	No

[a]The reduction in CPMG peak intensity upon addition of protein (n = 1)
[b]The net recovery of peak intensity upon addition of the $TIN2_{TBM}$ peptide to the compound-plus-protein sample (n = 1)

Supplementary Table 2: Analysis of fragment hits from the crystallographic screens carried out against TRF1$_{TRFH}$.

P3 XChem screen

Number:	Dataset no.	Structure	Crystal structure	Resolution (Å)	Buster ligand occupancy (%)*	Solubility (µM)	CPMG reduction (%)
**1**	TRF1-x0077	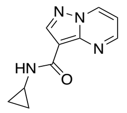	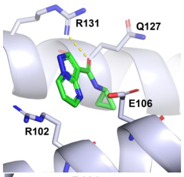	2.23	76	600	13
**2**	TRF1-x0197	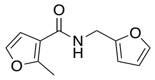	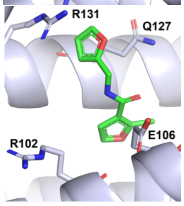	2.23	74	555	0
**3**	TRF1-x0276	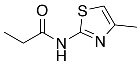	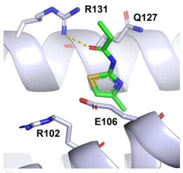	2.38	70	670	12
**4**	TRF1-x0284	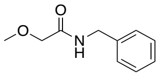	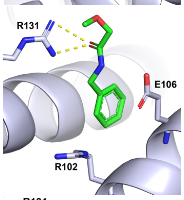	2.19	83	600	0
**5**	TRF1-x0289	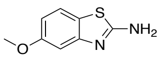	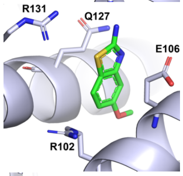	1.9	77	580	25
**6**	TRF1-x0323	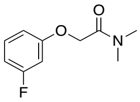	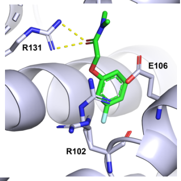	2.05	89	720	0
**7**	TRF1-x0325	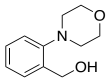	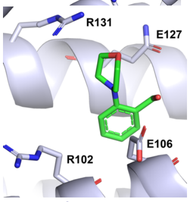	2.29	88	730	0
**8**	TRF1-x0348	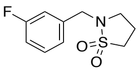	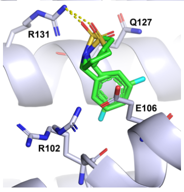	2.17	34/65	660	0
**9**	TRF1-x0394	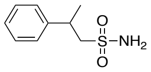	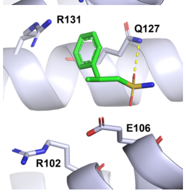	2.15	73	690	21

**10**	TRF1-x0439	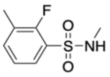	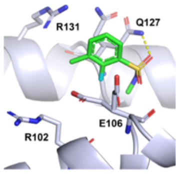	2.04	84	680	20
**11**	TRF1-x0560	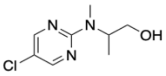	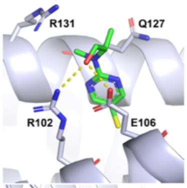	2.06	93	820	6
**12**	TRF1-x0586	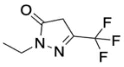	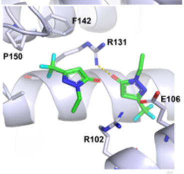	1.87	76/81	660	1
**13**	TRF1-x0648	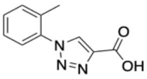	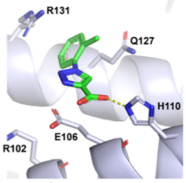	2.16	72	660	23
**14**	TRF1-x0656	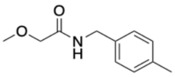	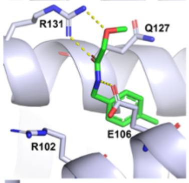	2.02	87	660	0
**15**	TRF1-x0674	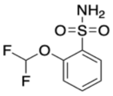	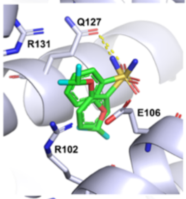	2.48	53/47	570	12
**16**	TRF1-x0713	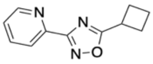	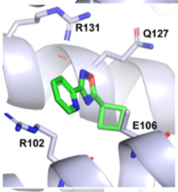	2.12	69	630	22
**17**	TRF1-x0715	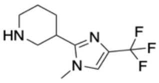	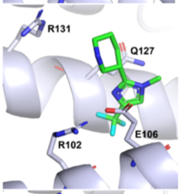	2.44	79	-	0
**18**	TRF1-x0741	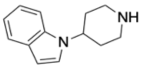	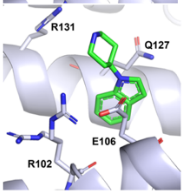	2.24	88	-	31
**19**	TRF1-x0845	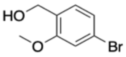	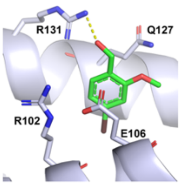	2.23	92	-	37

*Two values are shown in cases where two ligand conformations are observed in the binding site

P4 XChem screen

Number:	Dataset no.	Structure	Crystal structure	Resolution (Å)	Buster ligand occupancy (%)	Solubility (μM)	CPMG reduction (%)
**1**	TRF1_2-x0021	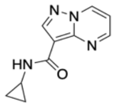	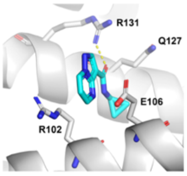	1.87	89	600	13
**6**	TRF1_2-x0612	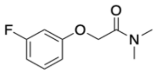	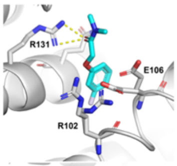	1.55	43	725	12
**15**	TRF1_2-x0076	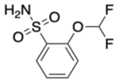	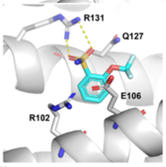	1.70	46	570	0
**16**	TRF1_2-x0425	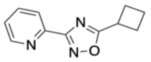	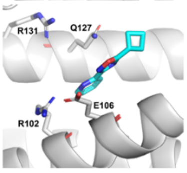	2.08	95	630	22
**45**	TRF1_2-x0009	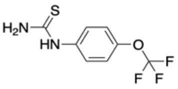	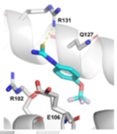	2.32	71	650	
**46**	TRF1_2-x0025	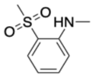	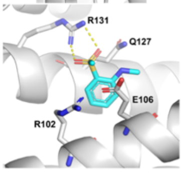	1.93	53	610	36
**47**	TRF1_2-x0116	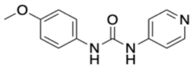	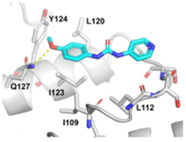	1.96	80	>1000	56
**48**	TRF1_2-x0195	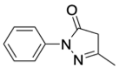	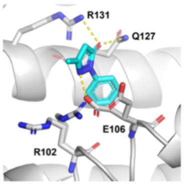	1.93	47	670	0
**49**	TRF1_2-x0238	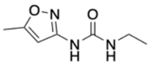	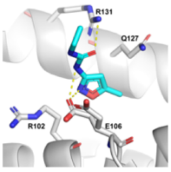	2.20	98	870	24

**50**	TRF1_2-x0292	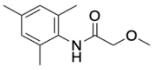	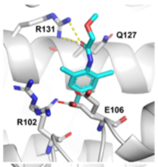	1.70	88	660	23
**51**	TRF1_2-x0312	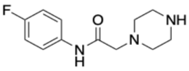	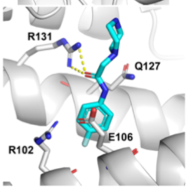	2.07	86	570	11
**52**	TRF1_2-x0522	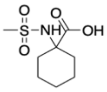	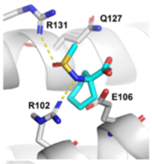	2.08	92	0	-
**53**	TRF1_2-x0572	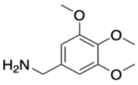	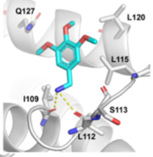	1.69	67	575	11
**54**	TRF1_2-x0592	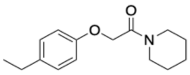	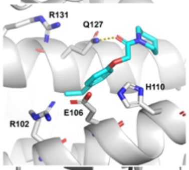	1.93	62	485	21
**55**	TRF1_2-x0656	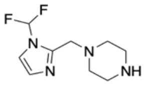	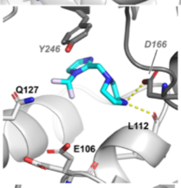	1.60	48	485	0
**56**	TRF1_2-x0866	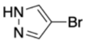	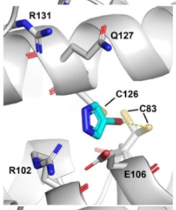	2.17	39		14
**57**	TRF1_2-x0869	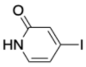	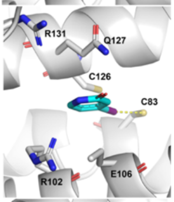	2.50	48	>1000	25
**58**	TRF1_2-x0874	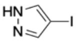	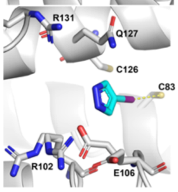	2.10	66	-	9
**59**	TRF1_2-x1043	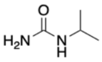	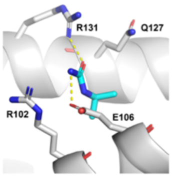	1.73	100	-	-

Supplementary Table 3: X-ray crystallography data collection and refinement statistics. Fragment hits from the first crystallographic fragment screen using the P$3_1$21 TRF1$_{TRFH}$ crystal system are numbered **1 – 19**. Three fragment hits developed from the LO-NMR fragment screen and soaked into TRF1$_{TRFH}$ crystals in the P$4_1 2_1$2 space group are numbered **27**, **32**, and **40.** Fragment hits from the second crystallographic screen using the P$4_1 2_1$2 TRF1$_{TRFH}$ crystal system are numbered **45 – 59**.

**Compound_ID**	**1**	**2**	**3**	**4**	**5**	**6**	**7**	**8**	**9**	**10**
**PDB_ID**	**9HF6**	**9HHK**	**9HF7**	**9HF8**	**9HF9**	**9HFA**	**9HFB**	**9HFC**	**9HFD**	**9HFE**
**Dataset_ID**	TRF1-x0077	TRF1-x0197	TRF1-x0276	TRF1-x0284	TRF1-x0289	TRF1-x0323	TRF1-x0325	TRF1-x0348	TRF1-x0394	TRF1-x0439
**Crystal**										
Space group	P 31 2 1	P 31 2 1	P 31 2 1	P 31 2 1	P 31 2 1	P 31 2 1	P 31 2 1	P 31 2 1	P 31 2 1	P 31 2 1
Unit cell dimensions (a/b/c in Å)	84.71, 84.71, 91.44	85.09, 85.09, 91.45	85.01, 85.01, 91.29	85.3, 85.3, 91.55	85.61, 85.61, 90.83	85.54, 85.54, 91.35	85.25, 85.25, 91.19	84.69, 84.69, 91.51	85.37, 85.37, 91.64	85.37, 85.37, 91.26
Unit cell angles (α/β/γ in °)	90, 90, 120	90, 90, 120	90, 90, 120	90, 90, 120	90, 90, 120	90, 90, 120	90, 90, 120	90, 90, 120	90, 90, 120	90, 90, 120
**Data collection and processing**										
Beamline	i04-1	i04-1	i04-1	i04-1	i04-1	i04-1	i04-1	i04-1	i04-1	i04-1
Wavelength (Å)	0.9179	0.9179	0.9179	0.9179	0.9179	0.9179	0.9179	0.9179	0.9179	0.9179
Integration program	XDS	XDS	XDS	XDS	XDS	XDS	XDS	XDS	XDS	XDS
Reduction program	AIMLESS	AIMLESS	AIMLESS	AIMLESS	AIMLESS	AIMLESS	AIMLESS	AIMLESS	AIMLESS	AIMLESS
Resolution range	42.35 - 2.23	73.69 - 2.23	57.3 - 2.23	57.49 - 2.19	45.39 (1.90)	45.68 - 2.05	57.38 - 2.29	57.23 - 2.17	45.82 - 2.15	45.63 - 2.04
Number of unique reflections [a]	18969 (1729)	19097 (1727)	19057 (1734)	20300 (1729)	30774 (1941)	24733 (1885)	17716 (1714)	20560 (1764)	21466 (1815)	24971 (1904)
Completeness [a]	100 (100)	100 (100)	100 (100)	100 (100)	100 (100)	100 (100)	100 (100)	100 (100)	100 (100)	100 (100)
Redundancy [a]	9.7 (9.7)	9.9 (9.9)	9.4 (9.4)	9.8 (9.3)	9.8 (8.7)	9.6 (9.9)	9.6 (10.1)	9.8 (9.4)	9.7 (9.5)	9.8 (10.1)
R_{merge} (%) [a]	0.069 (2.494)	0.084 (2.608)	0.147 (5.052)	0.066 (2.636)	0.071 (2.838)	0.078 (2.500)	0.095 (2.586)	0.064 (2.405)	0.102 (2.490)	0.062 (2.595)
I/σ(I) [a]	14.6 (0.7)	11.2 (1.0)	6.0 (0.3)	14.0 (0.7)	12.8 (0.6)	13.3 (0.7)	11.6 (1.0)	14.1 (0.6)	8.9 (0.7)	16.1 (0.7)
$CC_{1/2}$ [a, b]	1 (0.817)	0.998 (0.816)	0.998 (0.860)	0.999 (0.789)	0.999 (0.785)	1.000 (0.767)	0.999 (0.758)	0.976 (0.918)	0.998 (0.944)	1.000 (0.652)
**Refinement**										
Programme	BUSTER	BUSTER	BUSTER	BUSTER	BUSTER	BUSTER	BUSTER	BUSTER	BUSTER	BUSTER
R_{work} (%)	0.2291	0.2325	0.2342	0.2279	0.2131	0.2251	0.2292	0.2366	0.2293	0.2203
R_{free} (%)	0.2483	0.2458	0.2614	0.255	0.2279	0.2456	0.2652	0.2432	0.2446	0.2471
Number of residues	193	202	198	197	198	192	193	202	196	194
Number of water molecules	57	43	53	42	124	90	57	50	64	90
Average B-factor ($Å^2$)	84.2	84.11	84.34	82.58	59.09	63.42	76.47	88.99	74.09	67.48
Ramachandran favoured (%)	99.46	98.47	98.94	99.47	98.95	98.92	100	98.98	99.46	99.46
Ramachandran outliers (%)	0	0	0	0	0	0	0	0	0	0
RMSD bonds (Å)	0.012	0.0111	0.0121	0.012	0.0135	0.0133	0.0116	0.0124	0.0173	0.0119
RMSD angles (°)	1.367	1.313	1.317	1.31	1.336	1.33	1.334	1.322	1.392	1.3

[a]Values in parentheses refer to the highest resolution shell.

**Compound_ID**	**11**	**12**	**13**	**14**	**15**	**16**	**17**	**18**	**19**
**PDB_ID**	**9HFF**	**9HCL**	**9HLT**	**9HFG**	**9HLR**	**9HFH**	**9HFI**	**9HLP**	**9HLU**
**Dataset_ID**	TRF1-x0560	TRF1-x0586	TRF1-x0648	TRF1-x0656	TRF1-x0674	TRF1-x0713	TRF1-x0715	TRF1-x0741	TRF1-x0845
**Crystal**									
Space group	P 31 2 1	P 31 2 1	P 31 2 1	P 31 2 1	P 31 2 1	P 31 2 1	P 31 2 1	P 31 2 1	P 31 2 1
Unit cell dimensions (a/b/c in Å)	86.46, 86.46, 91.79	85.92, 85.92, 91.11	85.61, 85.61, 91.03	85.79, 85.79, 91.42	85.02, 85.02, 91.62	84.91, 84.91, 91.28	84.87, 84.87, 92.11	85.2, 85.2, 91.1	85.82, 85.82, 91.16
Unit cell angles (α/β/γ in °)	90, 90, 120	90, 90, 120	90, 90, 120	90, 90, 120	90, 90, 120	90, 90, 120	90, 90, 120	90, 90, 120	90, 90, 120
**Data collection and processing**									
Beamline	i04-1	i04-1	i04-1	i04-1	i04-1	i04-1	i04-1	i04-1	i04-1
Wavelength (Å)	0.9179	0.9179	0.9179	0.9179	0.9179	0.9179	0.9179	0.9179	0.9179
Integration program	XDS	XDS	XDS	XDS	XDS	XDS	XDS	XDS	XDS
Reduction program	AIMLESS	AIMLESS	AIMLESS	AIMLESS	AIMLESS	AIMLESS	AIMLESS	AIMLESS	AIMLESS
Resolution range	45.9 - 2.06	74.41 - 1.87	57.49 - 2.16	45.71 - 2.02	57.39 - 2.48	73.54 - 2.12	57.45 - 2.44	57.34 - 2.24	57.60 - 2.23
Number of unique reflections [a]	25010 (1906)	32610 (2043)	21166 (1799)	26013 (1892)	13996 (1552)	22083 (1796)	14711 (1612)	18844 (1703)	19399 (1763)
Completeness [a]	100 (100)	100 (100)	100 (100)	100 (100)	100 (100)	100 (100)	100 (100)	100 (100)	100 (100)
Redundancy [a]	9.6 (9.9)	9.8 (8.3)	9.9 (9.5)	9.7 (10.1)	9.6 (10.1)	9.7 (9.8)	9.9 (10.3)	9.9 (10.1)	9.7 (9.8)
R_{merge} (%) [a]	0.092 (1.901)	0.050 (1.791)	0.067 (1.920)	0.076 (2.583)	0.100 (1.643)	0.073 (2.569)	0.051 (2.370)	0.060 (2.592)	0.084 (1.889)
I/σ(I) [a]	9.8 (0.8)	19.7 (0.8)	13.3 (0.8)	13.6 (0.8)	8.5 (0.7)	12.4 (0.8)	17.3 (0.7)	14.7 (0.9)	12.2 (0.7)
$CC_{1/2}$ [a, b]	0.999 (0.794)	1.000 (0.728)	0.948 (0.963)	1.000 (0.702)	0.997 (0.855)	1.000 (0.960)	1.000 (0.695)	0.999 (0.858)	0.998 (0.878
**Refinement**									
Programme	BUSTER	BUSTER	BUSTER	BUSTER	BUSTER	BUSTER	BUSTER	BUSTER	BUSTER
R_{work} (%)	0.2238	0.2044	0.237	0.2282	0.2347	0.2286	0.2434	0.2356	0.2425
R_{free} (%)	0.2612	0.2203	0.2605	0.2525	0.2689	0.2628	0.2758	0.2551	0.2794
Number of residues	201	199	197	192	202	195	197	196	190
Number of water molecules	99	193	65	134	28	72	21	60	55
Average B-factor ($Å^2$)	68.44	54.24	79.67	62.15	101.15	74.79	108.81	90.98	79.37
Ramachandran favoured (%)	98.46	98.96	99.47	98.91	98.48	99.46	98.95	97.88	97.27
Ramachandran outliers (%)	0	0	0	0	0	0	0	0	0
RMSD bonds (Å)	0.0121	0.0126	0.0121	0.0132	0.0101	0.012	0.0125	0.0118	0.0119
RMSD angles (°)	1.344	1.337	1.3	1.32	1.231	1.313	1.345	1.345	1.372

[a]Values in parentheses refer to the highest resolution shell.

**Compound_ID**	**27**	**32**	**40**	**45**	**1**	**46**	**15**	**47**	**48**	**49**	**50**
**PDB_ID**	**9HD3**	**9HF4**	**9HD2**	**9HCM**	**9HD9**	**9HCN**	**9HCP**	**9HCQ**	**9HCR**	**9HCS**	**9HCT**
**Dataset_ID**	-	-	-	TRF1_2-x0009	TRF1_2-x0021	TRF1_2-x0025	TRF1_2-x0076	TRF1_2-x0116	TRF1_2-x0195	TRF1_2-x0195	TRF1_2-x0292
**Crystal**											
Space group	P 41 21 2	P 41 21 2	P 41 21 2	P 41 21 2	P 41 21 2	P 41 21 2	P 41 21 2	P 41 21 2	P 41 21 2	P 41 21 2	P 41 21 2
Unit cell dimensions (a/b/c in Å)	51.82, 51.82, 145.72	51.73, 51.73, 145.24	52.40 52.40 146.34	52.18, 52.18, 146.29	51.87, 51.87, 146.94	52.03, 52.03, 147.38	51.87, 51.87, 147.22	51.66, 51.66, 146.53	51.96, 51.96, 146.99	51.85, 51.85, 147.25	52.03, 52.03, 147.33
Unit cell angles (α/β/γ in °)	90, 90, 90	90, 90, 90	90, 90, 90	90, 90, 90	90, 90, 90	90, 90, 90	90, 90, 90	90, 90, 90	90, 90, 90	90, 90, 90	90, 90, 90
**Data collection and processing**											
Beamline	i03	i04	i04-1	i04-1	i04-1	i04-1	i04-1	i04-1	i04-1	i04-1	i04-1
Wavelength (Å)	0.9763	0.9537	0.9212	0.9212	0.9212	0.9212	0.9212	0.9212	0.9212	0.9212	0.9212
Integration program	XDS	XDS	XDS	XDS	XDS	XDS	XDS	XDS	XDS	XDS	XDS
Reduction program	AIMLESS	AIMLESS	AIMLESS	AIMLESS	AIMLESS	AIMLESS	AIMLESS	AIMLESS	AIMLESS	AIMLESS	AIMLESS
Resolution range	42.23 - 2.19	48.73 - 2.17	49.33 - 2.07	49.15 - 2.32	51.87 - 1.87	49.13 - 1.93	49.07 - 1.70	51.66 - 1.96	48.99 - 1.93	49.08 - 2.20	49.11 - 1.70
Number of unique reflections [a]	10947 (916)	11161 (929)	13216 (1003)	9421 (885)	17475 (1103)	16077 (1034)	23100 (1211)	15090 (1029)	15976 (1023)	10903 (911)	23251 (1210)
Completeness [a]	100 (100)	100 (100)	100 (100)	100 (100)	100 (100)	100 (100)	100 (100)	100 (100)	100 (100)	99.8 (99.9)	100 (100)
Redundancy [a]	25.4 (25.0)	24.2 (25.5)	14.1 (14.7)	13.9 (15.0)	13.5 (11.0)	13.8 (12.0)	12.9 (9.2)	14.1 (13.6)	13.1 (11.3)	13.9 (13.6)	13.1 (9.4)
R_{merge} (%) [a]	0.094 (2.820)	0.072 (2.602)	0.121 (2.255)	0.167 (2.612)	0.183 (1.576)	0.145 (2.949)	0.085 (1.900)	0.226 (2.854)	0.205 (2.789)	0.301 (2.427)	0.064 (2.443)
I/σ(I) [a]	19.0 (1.6)	20.1 (1.4)	8.8 (0.8)	10.5 (3.2)	7.6 (0.9)	11.2 (1.8)	13.4 (0.9)	8.0 (2.4)	8.8 (2.5)	13.0 (5.7)	15.9 (0.9)
$CC_{1/2}$ [a]	0.999 (0.833)	1.000 (0.927)	0.997 (0.855)	0.992 (0.977)	0.986 (0.839)	0.996 (0.845)	0.997 (0.713)	0.987 (0.945)	0.969 (0.853)	0.961 (0.446)	0.999 (0.708)
**Refinement**											
Program	BUSTER	BUSTER	BUSTER	BUSTER	BUSTER	BUSTER	BUSTER	BUSTER	BUSTER	BUSTER	BUSTER
R_{work} (%)	23.95	23.19	22.29	23.01	22.82	20.52	20.04	20.24	19.56	20.51	20.29
R_{free} (%)	27.38	25.75	26.78	31.2	26.99	22.74	24.44	24.53	21.68	24.23	24.04
Number of residues	206	204	205	205	204	205	205	204	204	204	204
Number of water molecules	26	22	48	48	79	99	129	100	134	100	117
Average B-factor ($Å^2$)	67.4	75.88	66.3	63.8	40.07	43.31	39.3	41.64	39.7	34.18	46.32
Ramachandran favoured (%)	99.5	96.98	99.5	99.5	97.5	98	98	98.49	97.99	97.5	97.5
Ramachandran outliers (%)	0	0	0	0	0	0	0	0	0	0	0
RMSD bonds (Å)	0.013	0.012	0.013	0.011	0.0131	0.0124	0.0135	0.013	0.0126	0.0119	0.0135
RMSD angles (°)	1.359	1.407	1.351	1.305	1.259	1.281	1.297	1.302	1.286	1.317	1.334

[a]Values in parentheses refer to the highest resolution shell.

Compound_ID	51	16	52	53	54	6	55	56	57	58	59
**PDB_ID**	**9HCU**	**9HCV**	**9HCW**	**9HCX**	**9HCY**	**9HCZ**	**9HD0**	**-**	**9HLQ**	**9HDA**	**9HD1**
**Dataset_ID**	TRF1_2-x0312	TRF1_2-x0425	TRF1_2-x0522	TRF1_2-x0572	TRF1_2-x0592	TRF1_2-x0612	TRF1_2-x0656	TRF1_2-x0866	TRF1_2-x0869	TRF1_2-x0874	TRF1_2-x1043
**Crystal**											
Space group	P 41 21 2	P 41 21 2	P 41 21 2	P 41 21 2	P 41 21 2	P 41 21 2	P 41 21 2		P 41 21 2	P 41 21 2	P 41 21 2
Unit cell dimensions (a/b/c in Å)	52.03, 52.03, 146.94	52.44, 52.44, 146.11	52.35, 52.35, 147.07	51.66, 51.66, 146.62	51.92, 51.92, 147.23	51.88, 51.88, 147.22	51.77, 51.77, 146.79	52.07, 52.07, 146.42	51.97, 51.97, 145.88	52.27, 52.27, 146.05	51.65, 51.65, 147.18
Unit cell angles (α/β/γ in °)	90, 90, 90	90, 90, 90	90, 90, 90	90, 90, 90	90, 90, 90	90, 90, 90	90, 90, 90	90, 90, 90	90, 90, 90	90, 90, 90	90, 90, 90
**Data collection and processing**											
Beamline	i04-1	i04-1	i04-1	i04-1	i04-1	i04-1	i04-1	i04-1	i04-1	i04-1	i04-1
Wavelength (Å)	0.9212	0.9212	0.9212	0.9212	0.9212	0.9212	0.9212	0.9212	0.9212	0.9212	0.9212
Integration program	XDS	XDS	XDS	XDS	XDS	XDS	XDS	XDS	XDS	XDS	XDS
Reduction program	AIMLESS	AIMLESS	AIMLESS	AIMLESS	AIMLESS	AIMLESS	AIMLESS	AIMLESS	AIMLESS	AIMLESS	AIMLESS
Resolution range	49.05 - 2.07	52.44 - 2.08	49.32 - 2.08	48.87 - 1.69	49.08 - 1.93	51.88 - 1.55	51.77 - 1.60	49.06 - 2.17	51.97 - 2.50	52.27 - 2.10	51.65 - 1.73
Number of unique reflections [a]	13101 (979)	13036 (968)	13072 (977)	22736 (1085)	15989 (1021)	30074 (1369)	27393 (1344)	10873 (527)	7528 (817)	12580 (989)	21763 (1144)
Completeness [a]	100 (99.9)	100 (100)	100 (100)	98.5 (94)	100 (100)	99.5 (94.2)	100 (100)	95.4 (100)	100 (100)	100 (100)	100 (100)
Redundancy [a]	12.6 (12.1)	14.2 (14.8)	14.2 (14.8)	12.1 (7.7)	13.7 (12.6)	11.5 (4.1)	12.0 (5.3)	9.9 (10)	13.3 (13.4)	14.2 (14.7)	13.3 (9.8)
R_{merge} (%) [a]	0.281 (2.779)	0.111 (2.938)	0.080 (2.411)	0.200 (2.134)	0.142 (2.409)	0.068 (1.202)	0.074 (1.298)	0.248 (6.329)	0.202 (3.003)	0.106 (2.796)	0.109 (2.729)
I/σ(I) [a]	5.8 (1.5)	10.9 (1.3)	14.2 (1.4)	6.5 (0.9)	9.5 (1.1)	15.1 (0.9)	16.0 (1.4)	8.8 (0.5)	6.8 (1.2)	13.9 (1.8)	13.9 (1.4)
$CC_{1/2}$ [a, b]	0.988 (0.837)	0.998 (0.867)	0.994 (0.777)	0.989 (0.621)	0.989 (0.685)	0.997 (0.773)	0.998 (0.701)	1.0 (0.4)	0.989 (0.645)	0.995 (0.810)	0.998 (0.491)
**Refinement**											
Program	BUSTER	BUSTER	BUSTER	BUSTER	BUSTER	BUSTER	BUSTER	BUSTER	BUSTER	BUSTER	BUSTER
R_{work} (%)	22.62	22.9	24.21	19.52	20.1	19.85	19.76	23.22	26.26	23.17	19.37
R_{free} (%)	25.99	26.84	25.53	24.71	22.28	21.43	22.44	28.91	29.74	28.29	22.66
Number of residues	204	204	204	204	204	204	206	204	204	206	204
Number of water molecules	78	49	34	133	126	153	127	58	13	42	143
Average B-factor (Å^2)	40.77	66.5	68.35	30.69	38.26	36.62	33.7	47.93	89.62	69.43	36.15
Ramachandran favoured (%)	97.99	99.5	97.49	98.49	97.99	97.99	98.51	97.99	97.41	97.03	99
Ramachandran outliers (%)	0	0	0	0	0	0	0	0	0	0.5	0
RMSD bonds (Å)	0.0122	0.0121	0.0119	0.0135	0.0124	0.0142	0.0138	0.012	0.0113	0.0122	0.0135
RMSD angles (°)	1.335	1.31	1.354	1.362	1.316	1.362	1.445	1.421	1.345	1.384	1.324

[a]Values in parentheses refer to the highest resolution shell.

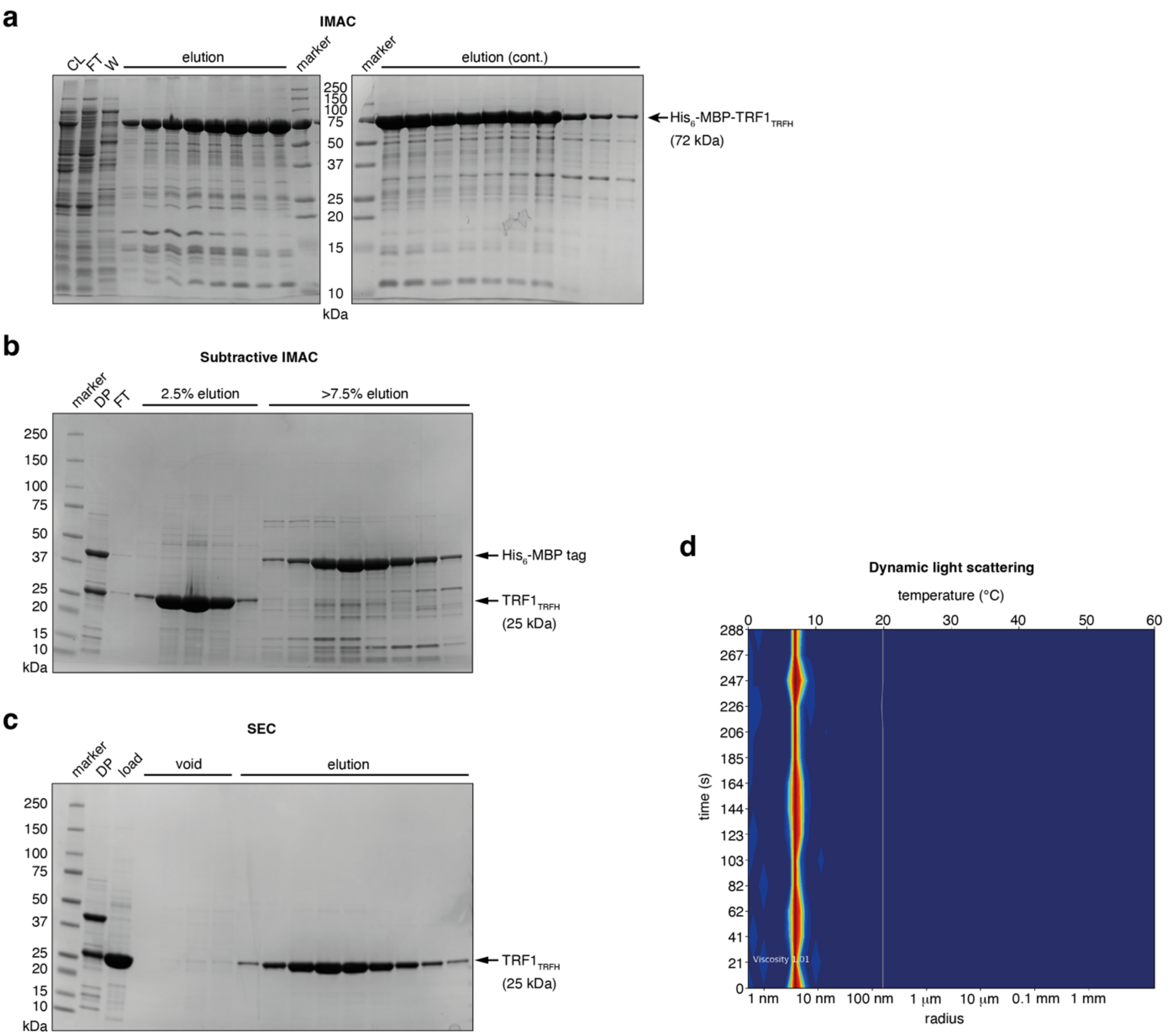

Supplementary Figure 1: TRF1$_{TRFH}$ purification and quality control. Samples were analysed by SDS-PAGE and Coomassie staining. **(a)** Immobilised metal ion affinity chromatography (IMAC) step: cell lysate (CL), flowthrough (FT), wash (W) and eluted protein. **(b)** Subtractive IMAC following 6xHis-MBP tag cleavage using TEV protease: dialysed and cleaved protein (DP), flowthrough (FT) and stepwise elutions of protein with 2.5% elution buffer and >7.5% elution buffer. **(c)** Final size exclusion chromatography (SEC) purification: dialysed and cleaved protein (DP), the protein loaded onto the column (load), the void volume peak and the elution peak. A cropped portion of panel (c) is shown in Fig. 2a. Theoretical molecular weights are indicated. **(d)** Dynamic light scattering (DLS) analysis of SEC-purified TRF1$_{TRFH}$ indicates homogeneity. The radius distribution plot shows the derived hydrodynamic radius (Rh) of the sample as a function of time, at a constant temperature. The colour scale indicates the relative abundance of each radius fraction, with blue to red representing the least abundant to most abundant species, respectively.

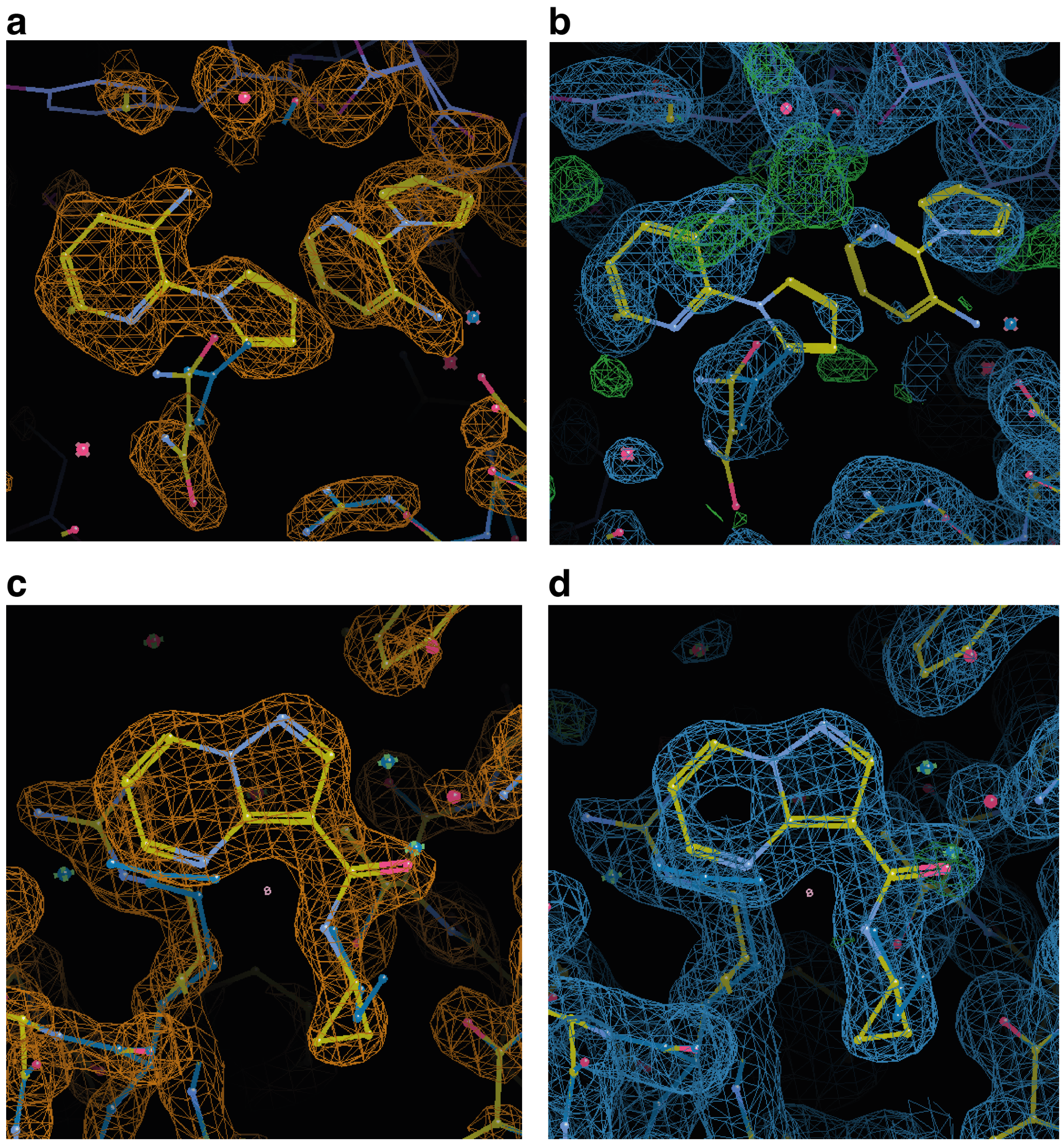

Supplementary Figure 2: Examples of PanDDA maps versus conventional 2mF$_o$-DF$_c$ electron density maps. (a) PanDDA map of dataset TRF1_2-x0455 showing two fragments bound to the TRF1$_{TRFH}$ crystal compared to **(b)** 2mF$_o$-DF$_c$ map after refinement with Buster. Given the poorly defined density, this hit was not taken forward. **(c)** PanDDA map of dataset TRF1_2-x0021 showing compound **1** bound to TRF1$_{TRFH}$ compared to **(d)** 2mF$_o$-DF$_c$ maps after refinement with Buster. All PanDDA maps are contoured at 2.40 RMSD level, and 2mF$_o$-DF$_c$ maps are contoured at 1.00 RMSD level.

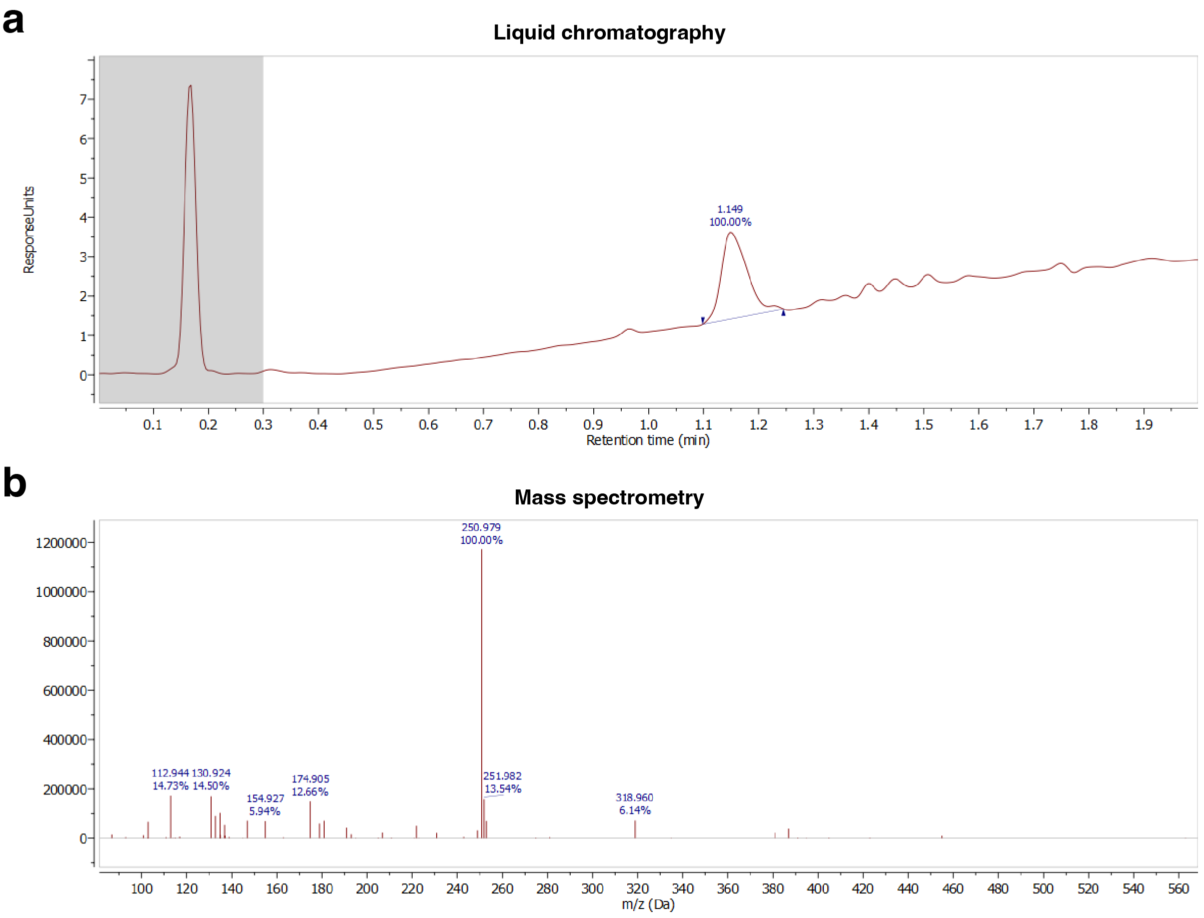

Supplementary Figure 3: Negative mode LC-MS analysis of compound 25. The LC-UV trace suggested a major compound eluting at 1.149 min **(a)** with an m/z value of 250.98 **(b)**. This value aligned with the molecular weight of compound **27** (251 g mol^{-1}), confirming that compound **25** in the original screening DMSO stock had degraded into compound **27**.

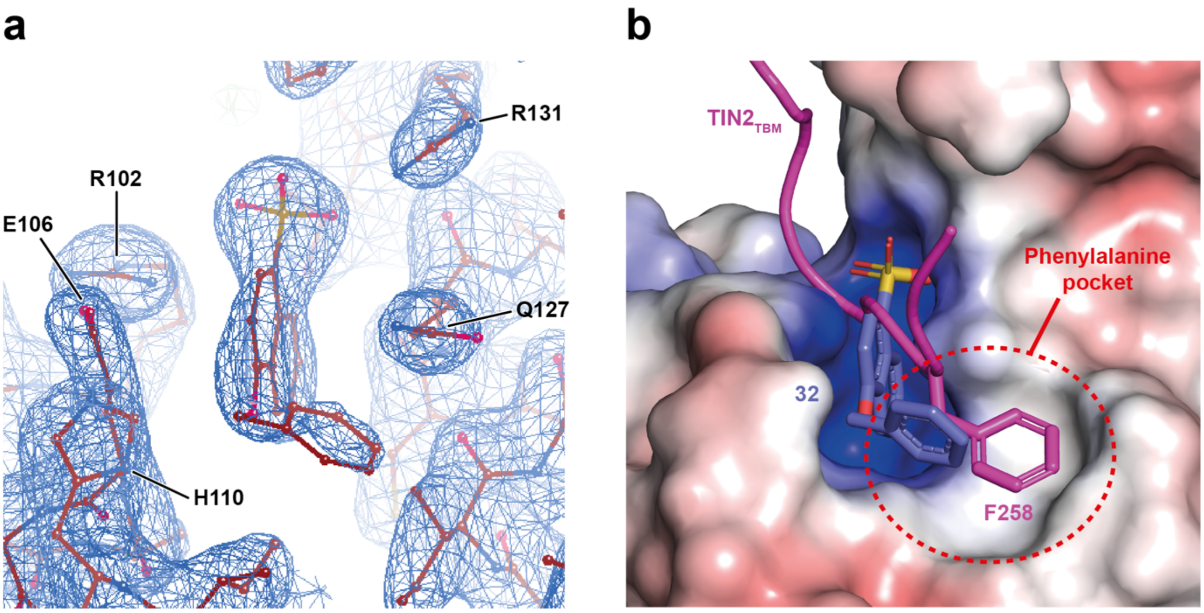

Supplementary Figure 4: Binding mode of compound 32. (a) Sigma-A weighted 2mF_o-DF_c electron density map of compound **32** bound to TRF1$_{TRFH}$ (from P$4_1 2_1 2$ crystals) contoured at an RMSD level of 1.0. **(b)** Phenylalanine of the TIN2$_{TBM}$ peptide (L258) from the TRF1:TIN2 co-crystal structure (PDB: 3BQO)[18] is shown in stick representation, coloured in magenta, superimposed onto the TRF1$_{TRFH}$:**32** structure, with compound **32** shown in stick representation in blue.

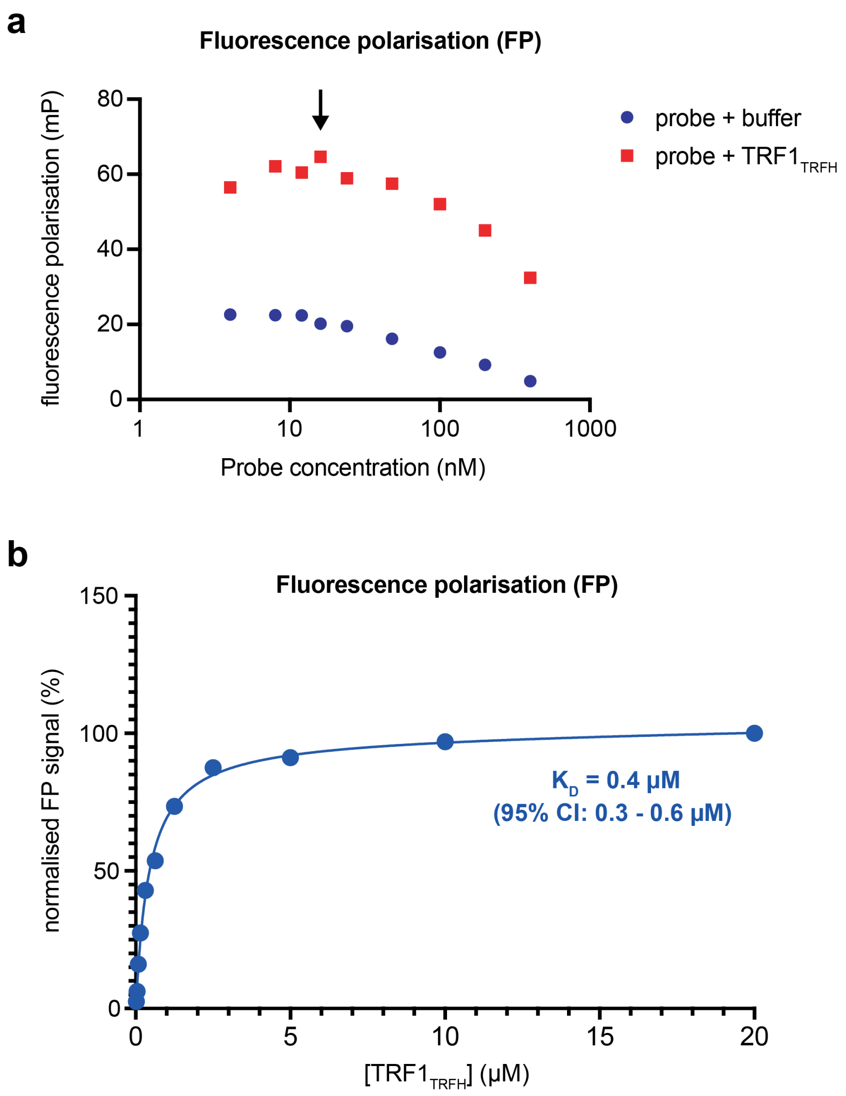

Supplementary Figure 5: Establishing conditions for an FP competition assay. (a) TIN2-FAM probe titration. The fluorescence polarisation (FP) signal was determined as a function of probe concentration both in the presence and absence of $TRF1_{TRFH}$. The black arrow indicates the probe concentration chosen for further experiments. **(b)** The FP signal of the TIN2-FAM probe as a function of $TRF1_{TRFH}$ domain concentration. Each data point is a mean value from two technical replicates of a single experiment. The calculated K_D with 95% CI values (profile likelihood) is indicated.

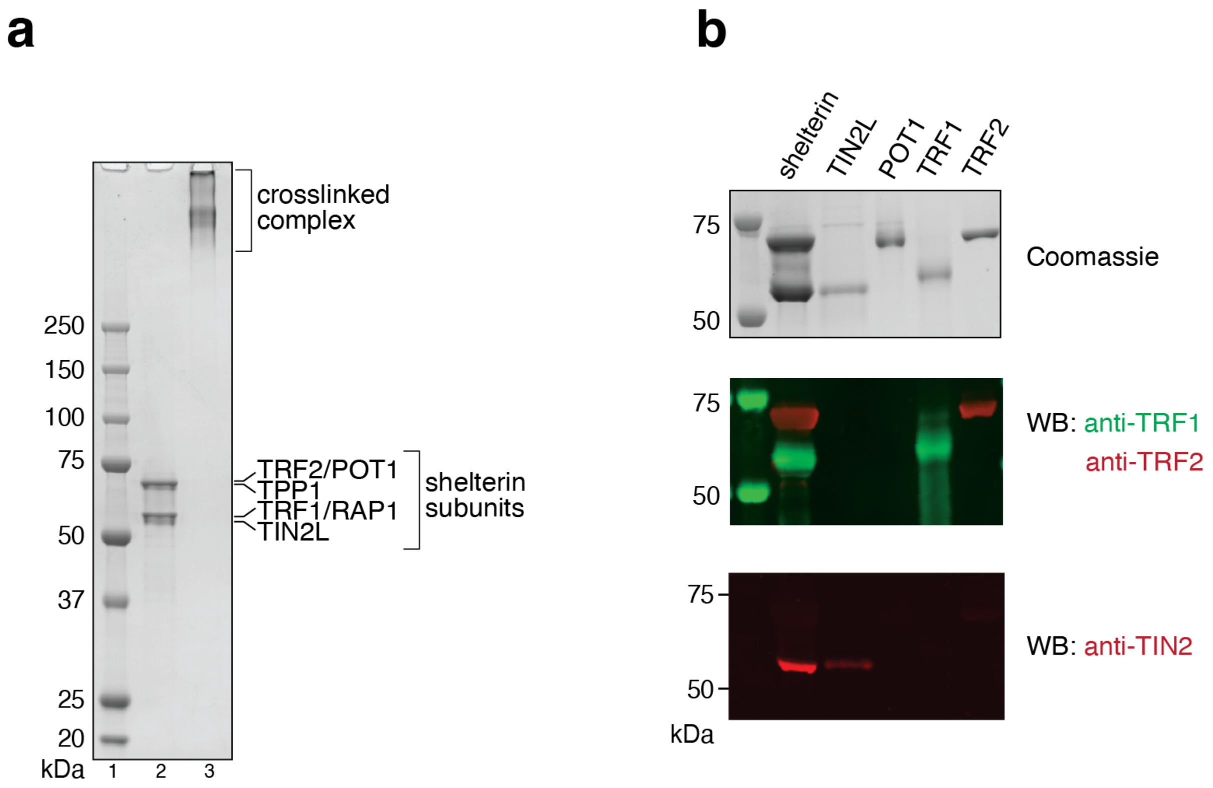

Supplementary Figure 6: SDS-PAGE/Coomassie and Western blot analysis of shelterin complex and subunits. (a) Subunit labels for the purified shelterin complex before crosslinking correspond to lane 2. Lane 3 shows the complex upon crosslinking by addition of 0.1% glutaraldehyde for 25 min at room temperature. **(b)** SDS-PAGE/Coomassie (top panel) and Western blot analysis of shelterin complex and subunits. Western blots are shown with anti-TRF1 signal in green, anti-TRF2 signal in red (middle panel) and anti-TIN2 antibody (bottom panel). Samples analysed (from left to right) are purified full shelterin complex, TIN2, POT1, TRF1 and TRF2. See Supplementary Fig. 9 for uncropped gel and Western blot images.

Mass photometry

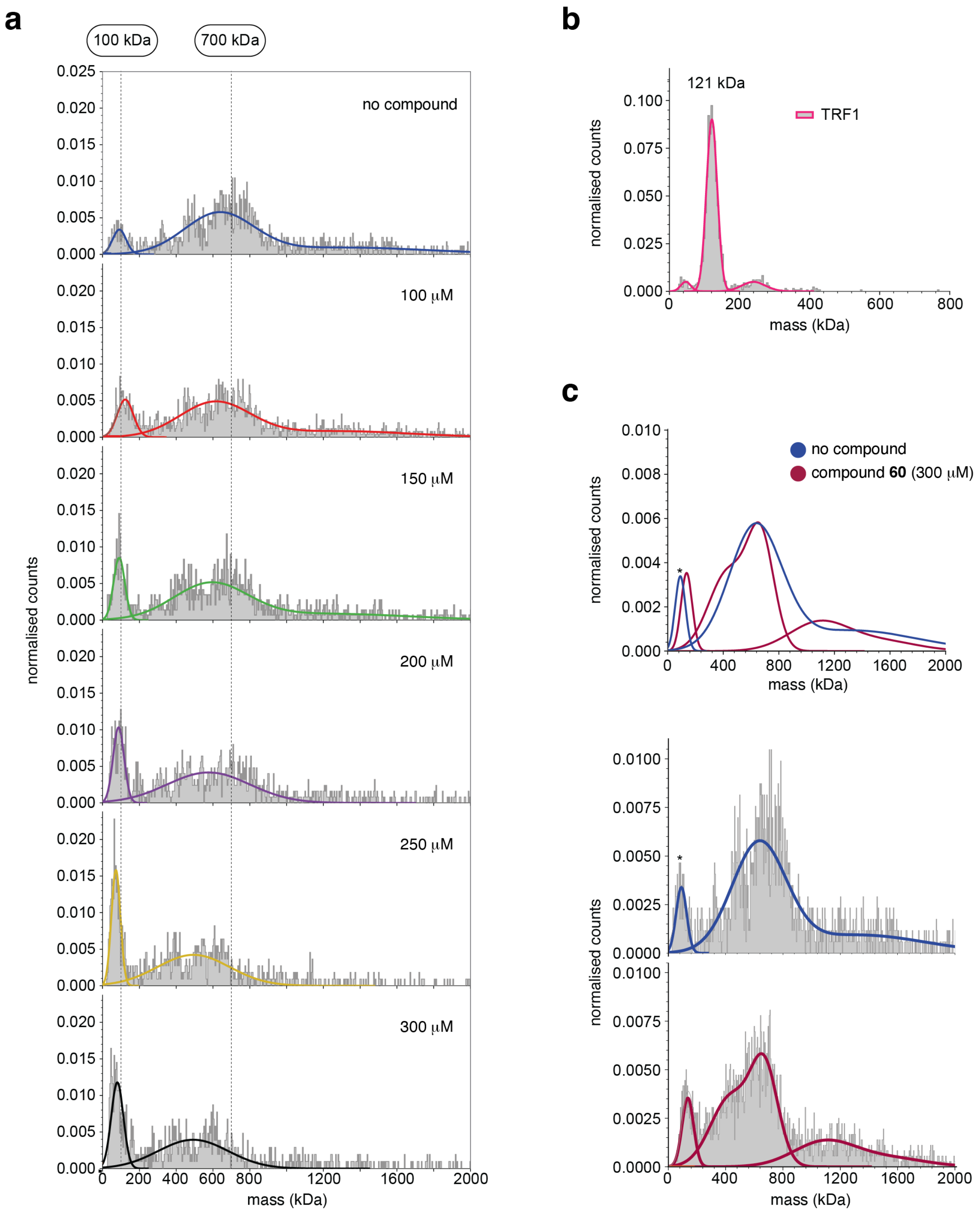

Supplementary Figure 7: Controls for mass photometry assay. (a) Mass distribution histograms for the Gaussian fits shown in the mass photometry assay (Fig. 6a). Gaussian fits are shown in colours corresponding to the main figure, and histograms are shown in grey**.** Mass photometry of **(b)** the full-length TRF1 sample and **(c)** shelterin with or without 300 µM of the inactive compound **60,** showing no eviction of TRF1 with the inactive compound**.** Top panel with individual Gaussian fits overlaid, bottom panel with mass distribution histograms and corresponding Gaussian fits. Asterisk (*) indicates a lower-molecular-weight contaminant in the shelterin-only sample. Gaussian fits also indicate the presence of higher-molecular-weight species in the sample.

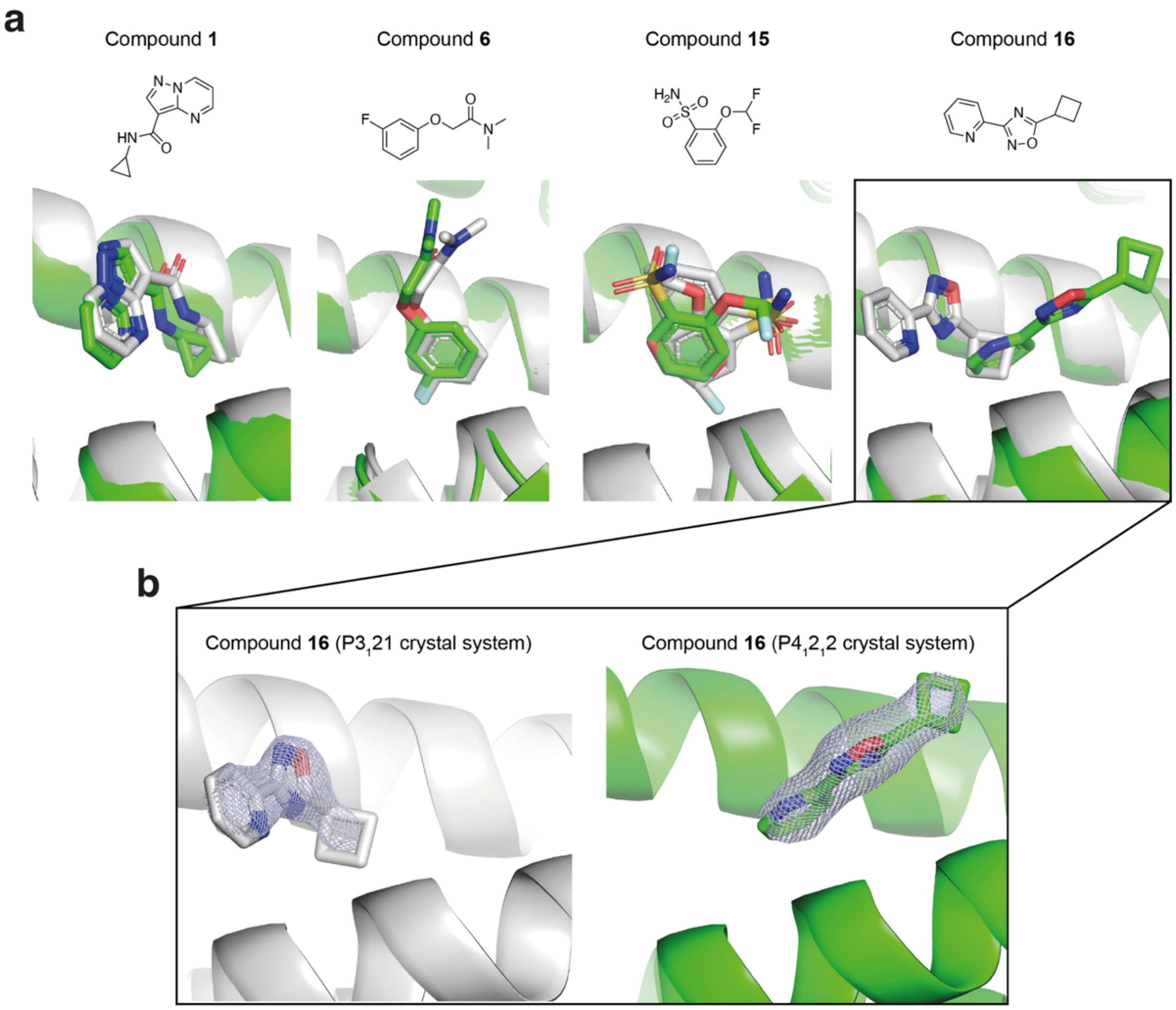

Supplementary Figure 8: Fragment hits common to both XChem screens. (a) Refined $TRF1_{TRFH}$:ligand structures showing the protein backbone in cartoon representation and bound ligands as sticks (coloured by heteroatom). Protein:ligand structural representations from $P3_121$ crystals are coloured in grey; those from $P4_12_12$ crystals are coloured in green. **(b)** Compound **16** bound to $TRF1_{TRFH}$ as per structures determined from $P3_121$ crystals and $P4_12_12$ crystals, in grey and green, respectively, with the Sigma-A weighted $2mF_o$-DF_c electron density maps of ligands contoured at an RMSD level of 0.8.

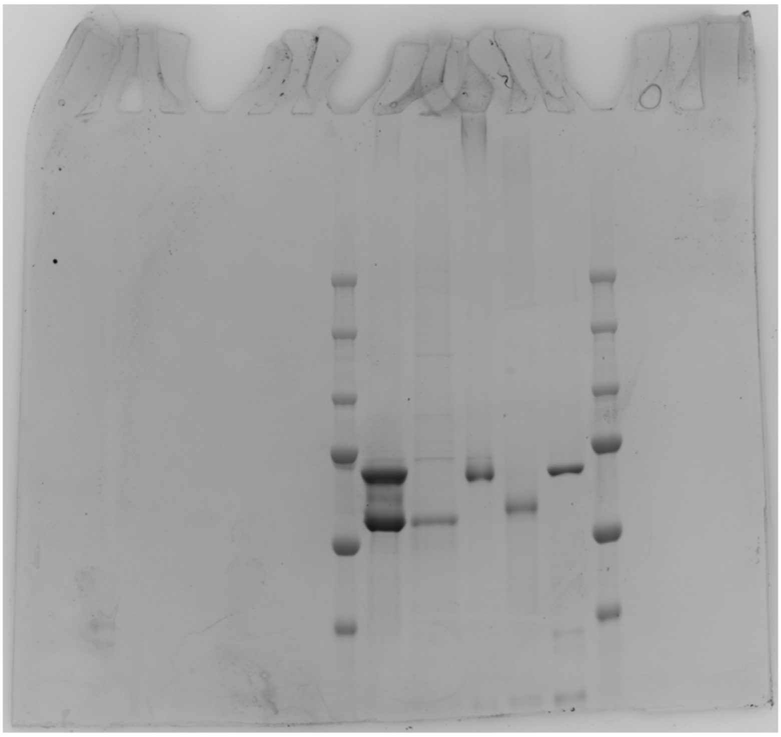

Coomassie

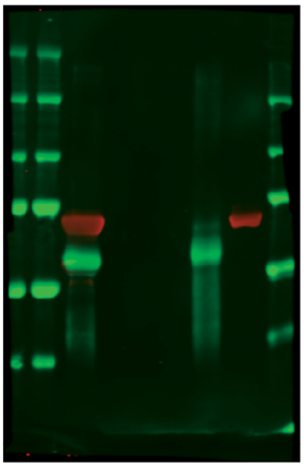

WB: anti-TRF1
anti-TRF2

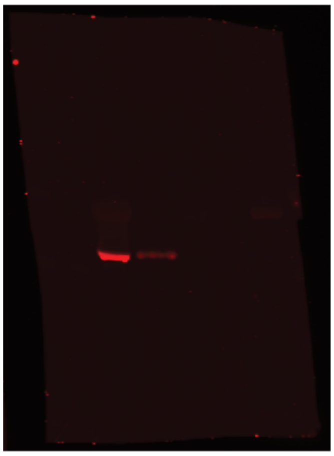

WB: anti-TIN2

Supplementary Figure 9: Images of uncropped gel and Western blots corresponding to Supplementary Fig. 6b.
